# Innovative, enhanced community management of non-hypoxaemic chest-indrawing pneumonia in 2–59-month-old children: a cluster-randomised trial in Africa and Asia

**DOI:** 10.1136/bmjgh-2021-006405

**Published:** 2022-01-05

**Authors:** EMPIC Study Group

**Keywords:** child health, paediatrics, pneumonia, cluster randomized trial, health policy

## Abstract

**Introduction:**

The WHO recommends oral amoxicillin for 2–59-month-old children with chest-indrawing pneumonia presenting at the health facility. Community-level health workers (CLHWs) are not allowed to treat these children when presented at the community level. This study aimed to evaluate whether CLHWs can safely and effectively treat children 2–59 months-old with chest indrawing with a 5-day course of oral amoxicillin in a few selected countries in Africa and Asia, especially when a referral is not feasible.

**Methods:**

We conducted a prospective multicountry cluster-randomised, open-label, non-inferiority trial in rural areas of four countries (Bangladesh, Ethiopia, India and Malawi) from September 2016 to December 2018. Children aged 2–59 months having parents/caregivers reported cough and/or difficult breathing presenting to a CLHW were screened for enrolment. CLHWs in the intervention clusters assessed children for hypoxaemia and treated non-hypoxaemic chest-indrawing pneumonia with two times per day oral amoxicillin (50 mg/kg body weight per dose) for 5 days at the community level. CLHWs in the control clusters identified chest indrawing and referred them to a referral-level health facility for treatment. Study supervisors performed pulse oximetry in the control clusters except in Bangladesh. Children were assessed for the primary outcome (clinical treatment failure) up to day 14 after enrolment. The accuracy and impact of pulse oximetry by CLHWs in the intervention clusters were also assessed.

**Results:**

In 208 clusters, 1688 CLHWs assessed 62 363 children with cough and/or difficulty breathing. Of these, 4013 non-hypoxaemic 2–59-month-old children with chest-indrawing pneumonia were enrolled. We excluded 116 children from analysis, leaving 3897 for intention-to-treat analysis. In the intervention clusters, 4.3% (90/2081) failed treatment, including five deaths, while in the control clusters, 4.4% (79/1816) failed treatment, including five deaths. The adjusted risk difference was -0.01 (95% CI −1.5% to 1.5%), which satisfied the prespecified non-inferiority criterion. CLHWs correctly performed pulse oximetry in 91.1% (2001/2196) of cases in the intervention clusters.

**Conclusions:**

The community treatment of non-hypoxaemic children with chest-indrawing pneumonia with 5-day oral amoxicillin by trained, equipped and supervised CLHWs is non-inferior to currently recommended facility-based treatment. These findings encourage a review of the existing strategy of community-based management of pneumonia.

**Trial registration:**

ACTRN12617000857303; The Australian New Zealand Clinical Trials Registry.

**Key questions**

**What is already known?**

- An estimated 22 million episodes of chest-indrawing pneumonia in children less than 5 years of age occur every year globally.
- Care-seeking for childhood pneumonia is low, and only 68% seek care from any source.
- The WHO’s integrated management of childhood illness chart booklet allows facility-based health worker to treat chest-indrawing pneumonia cases with a 5-day course of oral amoxicillin without referring them to a hospital.
- However, community-level health workers (CLHWs) are not allowed to treat children with chest-indrawing pneumonia with oral amoxicillin, leading to low coverage of pneumonia treatment.

**What are the new findings?**

- In the intervention clusters, CLHWs performed pulse oximetry and successfully treated non-hypoxaemic children 2–59-month-old with chest-indrawing pneumonia with oral amoxicillin, while in the control clusters, these children were referred to a higher-level health facility as per the standard management.
- The adjusted risk difference in the treatment failure rate in treatment failure rates between the intervention and control groups was −0.01%, which satisfied the prespecified non-inferiority criterion of the intervention to the control clusters.
- CLHWs correctly performed pulse oximetry in 91.1% of cases in the intervention clusters.

**Key questions**

**What do the new findings imply?**

- The community treatment of non-hypoxaemic children with chest -indrawing pneumonia with 5-day oral amoxicillin by trained, equipped, and supervised CLHWs is non-inferior to currently recommended facility-based treatment.
- CLHWs performed pulse oximetry correctly in most cases.
- These findings encourage a review of the existing strategy of community-based management of pneumonia.

## Introduction

Pneumonia is the number one killer of children 1–59-month-old globally, with over 800 000 pneumonia-related deaths every year.^1^ The burden of childhood pneumonia morbidity and mortality is higher in sub-Saharan Africa and South Asia than in other regions.^1 2^ The WHO and UNICEF developed integrated management of childhood illness (IMCI) for healthcare workers at primary care facility^3^ and integrated community case management (iCCM) protocol for community-level health workers (CLHWs).^4^ Both IMCI and iCCM protocols enable health workers to identify children with pneumonia using clinical signs such as respiratory rate and lower chest-indrawing.^3 4^ Globally, an estimated 138 million under-5 children had clinical pneumonia in 2015, and of these, 16% (22 million episodes) had chest-indrawing pneumonia.^2^

Several studies have shown the effectiveness of oral amoxicillin in treating chest-indrawing pneumonia in children 2–59 months of age.^5–8^ Based on this evidence, the WHO modified the pneumonia guidelines^9^ and subsequently IMCI chart booklet to recommend a 5-day course of oral amoxicillin in 2–59-month-old children with chest-indrawing pneumonia without referring them to a hospital.^3^ Two other trials supported the evidence after the WHO revision in pneumonia guideline.^10 11^ However, this recommendation was not extended to CLHWs using the iCCM protocol^4^ because of insufficient evidence on its safety and efficacy at the community level, as it was only available from two clinical trials, both from Pakistan.^12 13^ The only evidence from Africa was from an observational implementation research study that showed CLHWs could manage chest-indrawing pneumonia.^14^

Hypoxaemia is associated with a significantly increased risk of death in children with pneumonia.^15^ A pulse oximeter can non-invasively measure peripheral oxygen saturation (SpO_2_), and its use at the outpatient level has the potential to identify pneumonia with hypoxaemia for immediate referral for oxygen and injectable antibiotics to a hospital. The WHO recommends oxygen for <90% SpO_2_.^16^ The IMCI chart booklet recommends pulse oximeter use by a trained healthcare worker to assess hypoxaemia in children with cough and or difficulty breathing at a primary-level healthcare facility.^3^ However, the iCCM protocol does not recommend the use of a pulse oximeter by a CLHW.^4^

Improving appropriate care seeking from health facilities is an important part of IMCI.^3^ Prompt recognition of pneumonia signs and appropriate care seeking, which is essential to reduce pneumonia-related mortality in under-5 children, is a key component of the WHO/UNICEF Global Action Plan for the Control of Pneumonia and Diarrhoea framework.^17^ However, caregivers’ poor knowledge about symptoms and danger signs, faith in traditional care, distance to health facilities and cost can affect caregivers’ ability to seek proper and prompt care from health facilities.^18–21^ Care-seeking rates are inversely related to the distance to the closest health facility.^22 23^ Inappropriate care seeking, including traditional care, is common.^18 24^ Care seeking for children less than 5 years of age with respiratory symptoms is 68% globally.^25^ The role of CLHWs is crucial in improving the care seeking, providing prompt treatment and reducing pneumonia mortality in children, especially in low resource setting, where access to a health facility is difficult. Reduction in mortality, increasing care seeking and treatment for pneumonia, malaria and diarrhoea through CLHWs was recently demonstrated by the WHO Rapid Access Expansion iCCM programmed in the Democratic Republic of Congo, Malawi, Niger and Nigeria.^26–28^

High-quality data evaluating the management of chest-indrawing pneumonia by the CLHWs from various countries are essential before the iCCM protocol can be updated to make it consistent with the IMCI protocol. As hypoxaemia can accompany pneumonia, including the use of pulse oximetry will assist in the identification of patients with a high risk of mortality. We believe it will increase care seeking, reduce delayed referrals and provide timely and appropriate treatment, especially in hard to reach areas where access to health facilities is difficult and where referral is not feasible. This study aimed to gather evidence on whether CLHWs can safely and effectively treat non-hypoxaemic children 2–59-month-old with chest-indrawing with a 5-day course of oral amoxicillin in a few selected countries in Africa and Asia.

## Methods

### Trial design

We compared an enhanced management of pneumonia package with the standard management of pneumonia at the community level based on the iCCM protocol (box 1),^4^ using a cluster-randomised controlled, open-label, non-inferiority trial design. The trial was conducted in four countries (two in Africa (Ethiopia and Malawi) and two in Asia (Bangladesh and India)), which were selected due to a high burden of childhood pneumonia and having a functional iCCM programme to manage childhood pneumonia.

Box 1 **Definition of enhanced integrated community case management (pneumonia component) in intervention clusters^29^ and list of danger signs assessed in the study^4^**

**Part A: Community case management (pneumonia component) for community-level health workers**

I. Intervention clusters—enhanced community case management:

- Assess fast breathing, chest-indrawing and danger signs (as given below) in 2–59 month-old children.
- Perform pulse oximetry and refer hypoxaemic children to a hospital.
- Treat 2–59-month-old children with chest-indrawing with a 5-day course of oral amoxicillin.
  II. Control clusters—standard community case management
- Assess fast breathing, chest-indrawing and danger signs (as given below) in 2–59-month-old children.
- Refer children with chest-indrawing to a health facility.

**Part B: Danger signs evaluated in 2–59-month-old children screened for enrolment in the study**

- Cough for 14 days or more.
- Diarrhoea for 14 days or more.
- Blood in stools.
- Fever (temperature 38°C or above) for 7 days or more.
- Convulsions or fits.
- Difficult drinking or feeding or persistent vomiting.
- Unusually sleepy or unconscious.
- Severely malnourished as identified through mid-upper arm circumference <11.5 cm.
- Swelling of both feet.

### Participants

Children aged 2–59 months having parents/caregivers reported cough and/or difficult breathing presenting to a CLHW with chest-indrawing without any danger signs (box 1) were screened for enrolment. Difficult breathing was defined, and CLHWs were trained to identify any unusual pattern of breathing in their child as described by the caregivers, such as ‘fast’ or ‘noisy’ or ‘interrupted’ or ‘difficulty’ in breathing. We have published detailed procedures, implementation of iCCM and the characteristics of CLHWs country wise elsewhere.^29^ Briefly, in both intervention and control clusters, CLHWs identified 2–59-month-old children with chest-indrawing through visits by families to CLHWs. A standardised assessment of sick children per iCCM protocol was undertaken.^4^ CLHWs counted respiratory rate using a timer (UNICEF, Copenhagen, Denmark), observed chest-indrawing and excluded those with any danger sign (box 1). Children identified by the CLHW as having chest-indrawing pneumonia were re-examined by a study supervisor within half an hour to confirm the presence of chest-indrawing. Study supervisors were qualified nurses or clinical officers, hired and trained in the study methodology, supervision and pulse oximetry. In cases of disagreement, the supervisor’s measurements were considered final.

The CLHWs worked from 3 to 6 days every week at different sites and performed their work for around 5 to 8 hours on working days. Except for Bangladesh, the CLHWs who lived in the same communities could be accessed on the weekend or public holidays by the community members. A fair number of families would seek care from CLHWs in the study sites; however, a substantial proportion would seek direct care from the health centres or hospital or even private providers (at Asian sites) for various reasons, including unavailability of the CLHW or medicines at the community level or the perception of a child being sicker. For more details, see online supplemental Table 1.

10.1136/bmjgh-2021-006405.supp1 Supplementary data

### Interventions

In the control clusters, all enrolled children were immediately sent to a referral facility for further management as per the standard iCCM protocol,^4^ no study-mandated follow-up by the CLHWs was stipulated, who followed their routine practice, whereas, in the intervention clusters, enrolled children were treated by a CLHW with a 5-day course of oral amoxicillin dispersible tablets, 50 mg/kg per dose two times per day according to WHO age bands.^3^ On the day of enrolment (day 1), the first dose was given by the CLHW and demonstrated to the child’s parent/caregiver, and subsequent doses were administered by the parent/caregiver at home. If the child vomited within 30 min of administration of oral amoxicillin, the dose was readministered. The treating CLHWs followed these children on days 2, 4 and 7 after enrolment to assess for clinical improvement, need for referral and adherence to therapy.

CLHWs performed pulse oximetry in the intervention clusters to measure SpO_2_ using the Rad-5v pulse oximeter (Masimo, Irvine, California) on the great toe in smaller infants and toe or thumb in the older children. Study supervisors confirmed CLHWs’ SpO_2_ measurements to identify hypoxaemia (SpO_2_ <90%).^16^ Child was referred if the supervisor confirmed the presence of hypoxaemia. In the control group, study supervisors performed pulse oximetry to identify hypoxaemic children except for the Bangladesh site. Children of 2–59-month-old with chest-indrawing pneumonia, without danger signs, SpO_2_ ≥90%, and given informed written consent were enrolled in the trial.

All CLHWs in the control and the intervention clusters, study supervisors and outcome assessors were trained in iCCM^4 30^ through the programme’s existing training system. Also, CLHWs in intervention clusters were trained in pulse oximetry and treatment of children with oral amoxicillin. Hands-on refresher training was conducted by the investigators and study coordinators every 3–6 months. Study staff, including supervisors and independent outcome assessors, were also trained in pulse oximetry. Study supervisors and coordinators regularly validated the clinical skills (in both clusters) and pulse oximetry technique (in the intervention clusters only) of CLHWs, and those who performed poorly received hands-on training. Regular refresher training was also conducted at each site. Details of standardisation, supervision and monitoring process have been given elsewhere.^29^

Preparatory meetings with the Ministry of Health, district health officers and community leaders were held at each site before data collection. At the Indian site, CLHWs Accredited Social Health Activists (ASHA) who had not routinely treated children with fast breathing pneumonia as per standard iCCM protocol,^4^ were trained and evaluated for their ability to do so and to verify their acceptance in the community as treatment providers.^31^

### Study outcomes

The primary outcome was treatment failure by day 14. Treatment failure was defined as (a) death at any time up to day 14 of enrolment, or (b) clinical deterioration (defined as the presence of a danger sign or SpO_2_ <90%) on day 6, or (c) persistence of chest-indrawing on day 6 or (d) development of serious adverse event to amoxicillin by day 6 such as anaphylactic reaction, severe diarrhoea or generalised severe rash. The primary outcome was ascertained on day 6 (from number b to d) and day 14 (#a) of enrolment by independent outcome assessors (clinical officers/nurses trained in iCCM and trial methodology) who were unaware of the treatment received by the child to reduce potential measurement bias.^29^ The definitions of the clinical signs are given in online supplemental Table 2.

There were three components of the secondary outcome: (1) feasibility of using a pulse oximeter by CLHWs, (2) performance of CLHWs for using pulse oximetry against a standardised measurement by a trained study supervisor, (3) the impact of pulse oximetry on referral and treatment outcomes.^29^ However, in this paper, we only present the findings of the second and third components of the secondary outcome, and the results for the first outcome will be presented separately.

### Sample size

For sample size calculation, a 5000 population with a 3% birth rate was assumed per cluster, which gave 750 under 5 children at any point over a 2-year trial’s data collection period. Using a pneumonia incidence rate of 0.25 per child per year, 375 pneumonia cases per cluster per trial’s data collection period were estimated. Of these estimated cases, 15% were assumed as chest-indrawing pneumonia, leading to 50 children 2–59 months of age with chest-indrawing pneumonia per cluster during the trial’s data collection period. If there is truly no difference between the standard iCCM and enhanced iCCM (assuming 10% treatment failure in both groups based on previous research,^12 13^ with a design effect of 1.6 and attrition of 10%, then 1340 children 2–59 months of age with chest-indrawing pneumonia per group were required to be 90% sure that the upper limit of a two-sided 95% CI will exclude a difference in favour of the standard iCCM of >5%, assuming >50% difference in the treatment failure). We expected 30 cases per cluster over the data collection period, so 90 clusters (45 per group) were required for random allocation. A parallel trial in young infants up to 2 months with fast breathing pneumonia was also carried out at the same trial sites and at the same time, which required 99 clusters in each group.^32 33^ We chose the same number of clusters for this trial also, instead of the required 45 in each group.

### Randomisation

Clusters were randomly assigned in a 1:1 ratio to either standard management (control) or enhanced management (intervention) of pneumonia. Union in Bangladesh, Sub-centre in India and Health Centre in Ethiopia and Malawi were the randomisation units. We performed stratified randomisation using the cluster’s median or above population as the first stratum and below the median population as the second stratum. The computer-generated randomisation lists were prepared off-site by the WHO co-ordinating office in Geneva not involved with the study for each site. For Bangladesh, 52 clusters (26 intervention and 26 control) were randomly selected out of 54; two were dropped due to odd numbers in both strata. For Ethiopia, 20 clusters (10 intervention and 10 control) were randomly selected. For the India site, 92 clusters (46 intervention and 46 control) were randomly selected out of 95; three were dropped due to odd numbers in both strata. For Malawi, 44 clusters (22 intervention and 22 control) were randomly selected out of 46 health centres, two were dropped due to odd number in both strata (more details were published elsewhere).^29^

### Oversight and statistical methods

A technical steering committee and data safety and monitoring board (DSMB) provided oversight of the trial. Ethics approvals were obtained from the WHO Ethics Review Committee and the respective institutional ethics committees at each trial site. Informed written consent in the local language was obtained from the parent/caregiver and in the presence of a witness if the parent/caregiver was illiterate. The trial was performed by the principles of the Declaration of Helsinki.

Standard case report forms were used to collect data. All sites performed double data entry into a centralised database maintained by the Data Coordinating Centre (DCC). All sites uploaded a cleaned database to the DCC every month for quality checks. A planned interim analysis was conducted and reviewed by the DSMB after nearly 75% enrolment. As the enrolment rates were higher in Asian sites than African sites, the DSMB recommended ceasing enrolment at Asian sites (Bangladesh and India) in mid-2018 while continuing enrolment at African sites till the end of the year.

Descriptive statistics were used to calculate frequencies and percentages. Based on a predefined analysis plan to test for non-inferiority in the treatment failure rate between the intervention and control clusters, the difference in risk of treatment failure with a 95% CI was calculated and adjusted for sites and clusters. Post hoc prespecified subgroup analyses were performed to examine the difference in risk of treatment failure with 95% CI between intervention and control clusters.

For secondary outcomes, descriptive analyses were performed to evaluate the accuracy and impact of the use of pulse oximeter by CLHWs in intervention clusters. All analyses were performed using Stata V.14.2 (Stata-Corp, College Station, Texas).

### Role of the funding source

This study was funded by the Bill & Melinda Gates Foundation (#OPP1109076/INV-008068) through a grant to the WHO. The funders had no role in the study design or the collection, analysis or interpretation of the data. The funders did not write the report and had no role in the decision to submit the paper for publication.

### Patient and public involvement statement

The development of the research question was informed by the large burden of pneumonia-related mortality among children worldwide. Patients were not advisers in this study, nor were they involved in the design, recruitment or conduct of the study. Results of this study will be made publicly available through open-access publication where study participants may access them.

## Results

### Enrolment, patient flow and baseline characteristics

**Figure 1:**
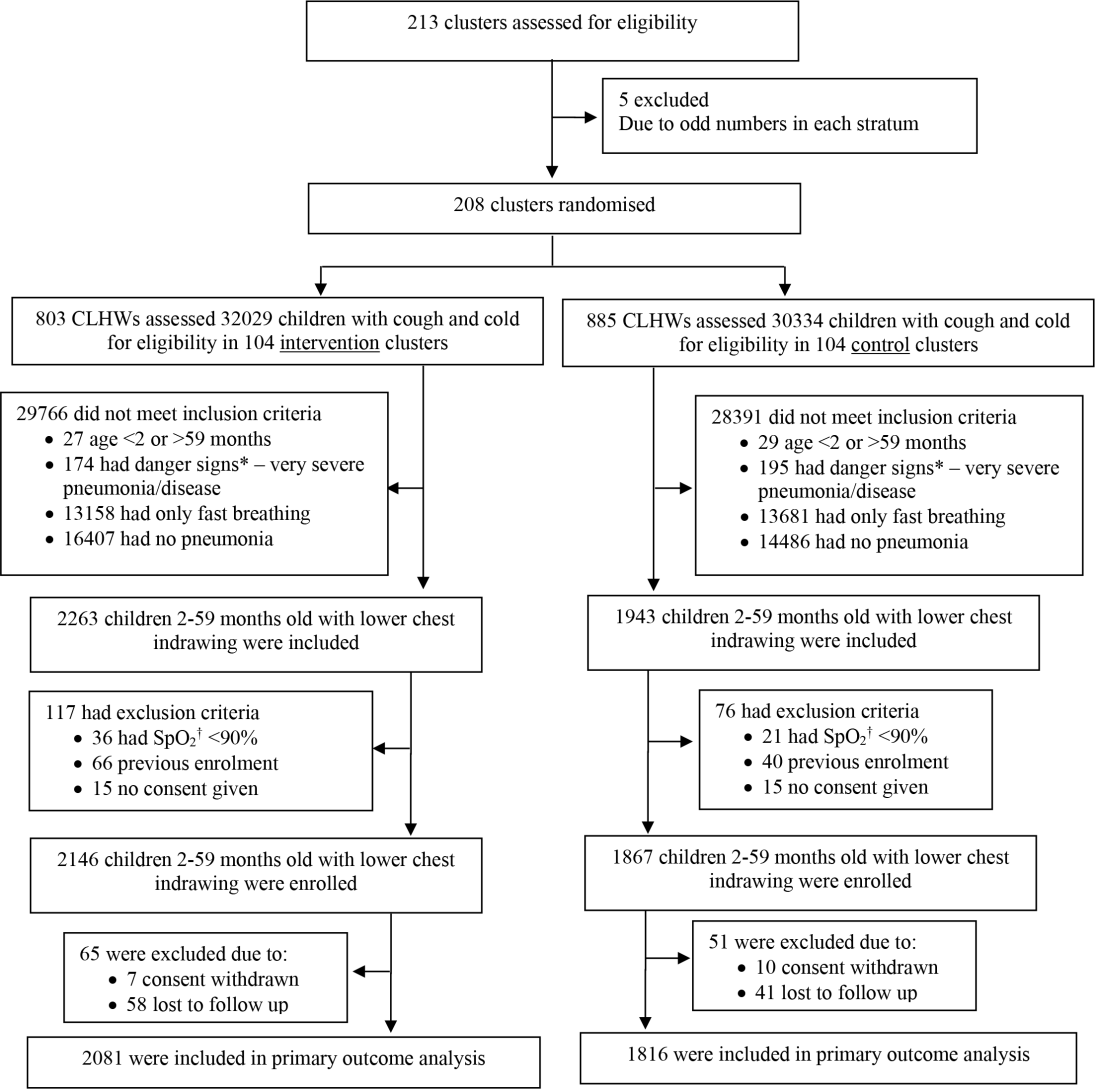
Screening, randomisation and follow-up.*Danger sign is defined as the presence of any of the following signs: cough for 14 days or more, diarrhoea for 14 days or more, blood in stools, fever (temperature 38°C or above) for 7 days or more, convulsions or fits, difficult drinking or feeding or persistent vomiting, unusually sleepy or unconscious, severely malnourished as identified through mid-upper arm circumference <11.5 cm, swelling of both feet. †SpO_2_: oxygen saturation. CLHW, community-level health worker.

CLHWs screened 62 363 children who presented with cough and/or difficulty in breathing at all four trial sites in both clusters, intervention (104 clusters and 32 029 children) and control (104 clusters and 30 334 children) (figure 1). Online supplemental Table 3 presents the distribution of danger signs identified by the CLHWs during screening. Of 4206 children, 2–59-month-old with chest-indrawing, 57 (1.4%) had hypoxaemia, 106 (2.5%) were previously enrolled and 30 (0.7%) did not give consent. We enrolled 2146 children in the intervention and 1867 in the control clusters. CLHWs followed 2113 (98.5%), 2090 (97.4%) and 1871 (87.2%) on day 2, 4 and 7 after enrolment in the intervention clusters (see online supplemental Table 4 for more details). Primary outcome data were available for 2081 children in intervention and 1816 in control clusters.

The data collection phase was carried out in Bangladesh between December 2016 and May 2018, Ethiopia between September 2016 and December 2018, India between March 2017 and May 2018 and Malawi between December 2016 and July 2017. There were no important differences between cluster and individual level baseline characteristics for the intervention and control clusters at all four sites (table 1).

**Table 1:** Characteristics of children at enrolment

	Intervention clusters	Control clusters
I. Cluster level characteristics		
**Bangladesh**		
Clusters—number	26	26
Children enrolled—number	556	393
Population size per cluster—median (IQR)	25 000 (21 000 to 30000)	25 000 (21 000 to 28000)
Community-level health workers per cluster—median (IQR)	13 (11, 14)	12 (10, 15)
Distance to referral facility per cluster in kilometres—median (IQR)	17 (10, 22)	12 (7, 20)
**Ethiopia**		
Clusters—number	10	10
Children enrolled—number	678	727
Population size per cluster—median (IQR)	24 000 (17 000 to 31000)	20 000 (13 000 to 27000)
Community-level health workers per cluster—median (IQR)	7 (4, 11)	7 (6, 10)
Distance to referral facility per cluster in kilometres—median (IQR)	37 (20, 47)	22 (10, 30)
**India**		
Clusters—number	46	46
Children enrolled—number	684	593
Population size per cluster—median (IQR)	10 000 (8000 to 13000)	10 000 (8000 to 13000)
Community-level health workers per cluster—median (IQR)	9 (8, 12)	10 (7, 12)
Distance to referral facility per cluster in kilometres—median (IQR)	17 (10, 24)	19 (14, 27)
**Malawi**		
Clusters—number	22	22
Children enrolled—number	228	154
Population size per cluster—median (IQR)	21 000 (17 000 to 26000)	21 000 (14 000 to 30000)
Community-level health workers per cluster—median (IQR)	6 (2, 9)	5 (3, 7)
Distance to referral facility per cluster in kilometres—median (IQR)	43 (24, 50)	54 (29, 60)
**II. Participant level characteristics**		
Number of children enrolled at all four sites	(N=2146)	(N=1867)
Age (months)—mean (SD)	15.4 (13.3%)	14.2 (12.4%)
Age 2–11 months – no. (%)	1120 (52.2%)	1059 (56.7%)
Age 12–59 months—number (%)	1026 (47.8%)	808 (43.3%)
Male sex—number (%)	1245 (58.0%)	1118 (59.9%)
Mid upper arm circumference (MUAC)* cm—mean (SD)	13.5 (1.2)	13.4 (1.2)
MUAC between 11.5 and 12.5 cm*—number (%)	358 (24.2%)	375 (29.8%)
Respiratory rate (breaths/minute)—mean (SD)	55.5 (9.3)	55.3 (9.0)
Respiratory rate ≥60 breaths/minute—number (%)	500 (27.5%)	518 (27.7%)
Axillary temperature (^o^C)—mean (SD)	37.1 (0.8)	37.2 (0.8)
Axillary temperature ≥38°C—number (%)	297 (13.8%)	315 (16.9%)

*Among children≥6 months of age.

### Primary outcome

In the intervention clusters, 90 (4.3%) children showed clinical treatment failure by day 14 after enrolment, including five deaths, while in the control clusters, 79 (4.4%) children failed treatment, including five deaths (table 2). The adjusted risk difference between the intervention and the control clusters was −0.01% (95% CI −1.5% to 1.5%). The subgroup analyses showed no difference in the risk of treatment failure in the intervention and control clusters. No serious adverse events of treatment were observed in either group. For more details on deaths, see online supplemental Table 5.

**Table 2:** Treatment failure rates in the two clusters

	Intervention (n=2081)	Control (n=1816)	Adjusted* risk difference (95% CI)
**Treatment failure† among all enrolled children – no. (%**)	**90** (**4.3%**)	**79** (**4.4%**)	−**0.01% (-1.5% to 1.5%**)
Reasons for treatment failure—number (%)			
Death at any time up to day 14 of enrolment	5 (0.2%)	5 (0.3%)	
Clinical deterioration on day 6‡	8 (0.4%)	11 (0.6%)	
Persistence of chest-indrawing on day 6	77 (3.7%)	63 (3.5%)	
Serious adverse event to amoxicillin by day 6	0 (0.0%)	0 (0.0%)	
**Treatment failure by subgroups**	**n/N (%)**	**n/N (%)**	
Region—number/total number (%)			
African sites	40/856 (4.7)	42/840 (5.0)	−0.4% (−3.0% to 2.1%)
Asian sites	50/1225 (4.1)	37/976 (3.8)	0.3% (−1.5% to 2.2%)
Age categories—number/total number (%)			
2–11 months	73/1079 (6.8)	62/1024 (6.1)	0.6% (−1.9% to 3.1%)
12–59 months	17/1002 (1.7)	17/792 (2.1)	−0.4% (−1.8% to 0.9%)
Sex—number/total number (%)			
Male	54/1205 (4.5)	52/1089 (4.8)	−0.3% (−2.2% to 1.6%)
Female	36/876 (4.1)	27/727 (3.7)	0.5% (−1.7% to 2.7%)
Mid upper arm circumference (MUAC)§—number/total number (%)			
MUAC between 11.5 and 12.5 cm	20/347 (5.8)	12/372 (3.2)	2.5% (−0.8% to 5.8%)
MUAC >12.5 cm	19/1079 (1.8)	24/848 (2.8)	−1.1% (−2.5% to 0.3%)
Respiratory rate (breaths per minute)—number/total number (%)			
<60 breaths/minute	54/1513 (3.6)	47/1323 (3.5)	0.1% (−1.5% to 1.6%)
≥60 breaths/minute	36/568 (6.3)	32/493 (6.5)	−0.1% (−3.2% to 3.1%)
Axillary temperature (^o^C)—number/total number (%)			
Axillary temperature <38°C	83/1800 (4.6)	64/1514 (4.2)	0.4% (−1.2% to 2.0%)
Axillary temperature ≥38°C	7/281 (2.5)	15/302 (5.0)	−1.8% (−5.4% to 1.8%)

*Adjusted for sites and clusters.

†Defined as (a) death any time up to 14 days of enrolment; or (b) Clinical deterioration defined as the presence of any danger signs [Unable to feed or poor feeding on observation, convulsion, unusually sleepy or unconscious, vomit everything] or SpO_2_ <90%; or (c) persistence of chest-indrawing on day 6; or (d) development of serious adverse event to amoxicillin by day 6.

‡Defined as the presence of any danger sign (unable to feed or poor feeding on observation, convulsion, unusually sleepy or unconscious, vomit everything) or SpO_2_ <90%.

§Among children≥6 months of age.

### Secondary outcome

In the intervention clusters, CLHWs performed pulse oximetry on 2255 children with chest-indrawing, and in 91.1% (2001/2196) of cases, they performed all steps of pulse oximetry assessment according to the instructions (table 3). The median (IQR) of SpO_2_ reading was 97% (94% and 99%). Compared with supervisors’ pulse oximeter readings, readings by CLHWs were either the same or within 2% of supervisors’ reading for 86.6% (1901/2196) of the patients. Thirty-six (1.6%) children had SpO_2_ <90% and were referred immediately to the hospital, and all of them survived after 14 days after the initial assessment. Online supplemental Table 6 presents the sitewise breakup of children in the intervention clusters with various SpO_2_ categories.

**Table 3:** Use of pulse oximetry by CLHWs in intervention clusters

A) Basic distribution of pulse oximetry measurements	Children with chest-indrawing
Pulse oximetry performed by CLHWs—number/ total number (%)	2255/2263 (99.6%)
**Oxygen saturation**—median (IQR)	
Overall	97 (94, 99)
Bangladesh	97 (96, 99)
Ethiopia	93 (91, 95)
India	98 (97, 100)
Malawi	97 (95, 99)
**Oxygen saturation category**—number/total number (%)	
<90%	36/2255 (1.6%)
90–<93%	355/2255 (15.7%)
93%–100%	1864/2255 (82.7%)
B) Secondary outcome	**Children with chest-indrawing**
Performance of CLHWs* for using pulse oximetry—number/total number (%)	
CLHWs performed all steps† as per instructions	2001/2196 (91.1%)
**The difference in SpO_2_‡ readings between CLHWs and supervisors**—number/ total number (%)	
No	951/2196 (43.3%)
1%	625/2196 (28.5%)
2%	325/2196 (14.8%)
3% or more	295/2196 (13.4%)
**Impact of pulse oximetry on referral and outcomes—number/total number (%)**	
Hypoxaemic children§ identified and referred to hospital	36/2255 (1.6%)
Alive after 14 days of initiation assessment	36/36 (100%)

*Among CLHWs whose pulse oximetry were validated by supervisors.

†All steps to perform pulse oximetry are: (i) cleaned the equipment before use, (ii) turned on the device correctly, (iii) selected the correct probe, (iv) attached the probe correctly, (v) positioned the child correctly and (vi) determined the reading correctly.

‡SpO_2_: Oxygen saturation.

§Defined as SpO_2_ <90%. CLHWs identified 32 hypoxaemic children, while the supervisor only identified four children. Supervisors readings were used to make clinical decisions and during analysis.

CLHW, community-level health worker.

### Adherence to treatment

Treatment adherence was good in the intervention clusters as 83.8% (1724/2058) of the patients received all 10 doses of amoxicillin for 5 days, and only 3.5% (73/2058) consumed less than 4 days of medicine (table 4). In the control clusters, 10.5% (167/1594) received hospitalised treatment, while 80.3% (1280/1594) received outpatient treatment.

**Table 4:** Compliance with recommended treatment strategy in enrolled children in intervention and control clusters

Intervention clusters*—5 day oral amoxicillin treatment (10 doses)—number (%)	Children with chest-indrawing (N=2058)
Received treatment for	
Full 5 days (10 doses)	1724 (83.8%)
4 to <5 days (8 to <10 doses)	261 (12.7%)
<4 days (<10 doses)	73 (3.5%)
**Control clusters—treatment at a health facility following referral**—number (%)	**Children with chest-indrawing (N=1594**)
Received	
Inpatient treatment in a hospital	167 (10.5%)
Outpatient treatment from any physician clinic/outpatient department of a hospital	1280 (80.3%)
Any other treatment	147 (9.2%)

*Missing data of 88 children were excluded from this analysis.

†Information was not collected from 273 children enrolled in the first few months of the study, who were excluded from this analysis.

## Discussion

### Principal findings

Our results demonstrate that 2–59-month-old non-hypoxaemic children with chest-indrawing, 5-day oral amoxicillin treatment by trained, supervised and equipped CLHWs were non-inferior to referral to a health facility for treatment. Children treated in the community by the CLHWs had a similar treatment failure rate to those referred to a health facility.

### Comparison with other literature

Two similar trials from Pakistan also showed that community-based treatment of chest-indrawing pneumonia was safe and effective.^12 13^ In the Haripur, Pakistan trial, children 2–59-month-old with chest-indrawing who received oral amoxicillin treatment from CLHWs had a significantly lower treatment failure rate (9%) than those who received the standard management of assessment and referred to a facility (18%).^12^ In the Matiari, Pakistan trial, children who received oral amoxicillin treatment from CLHWs had a non-significant lower treatment failure rate (8%) than those who received standard care (13%).^13^ The treatment failure rates in both these trials^12 13^ were higher than those reported in our trial. We believe that a few treatment failure criteria used in these trials, that is, presence of fever plus lower chest-indrawing on day 3 of treatment, presence of fever on day 6 and change of antibiotic,^12 13^ were not used in our trial. When we recalculated the treatment failure for the Haripur, Pakistan trial using our trial treatment failure definition, the treatment failure in the intervention group was 5.8% vs 8.6% in the control group (non-significant risk difference −2.8%). Also, we performed pulse oximetry to exclude hypoxaemic children, while no pulse oximetry was carried out in those trials.^12 13^ Similar to our study, these trials reported a low mortality rate (<1%) in both intervention and control groups.^12 13^ This could be attributed to the careful selection of study participants who did not have signs of very severe disease or other comorbidity and to the timely and appropriate treatment provided.

Few observational studies from community and health facility levels have reported lower chest-indrawing pneumonia from Africa.^14 34–37^ In a large community-based study in Homabay county, Kenya, CHWs were successfully able to treat children with lower chest-indrawing in the majority of cases and reported a low treatment failure rate (2.1%) and five deaths (0.3%), which are similar to our data.^14^ A pneumococcal vaccine study from Malawi introduced pulse oximeter use by health facility workers and CLHWs,^34 35^ which substantially increased the referral to the hospitals for pneumonia cases, especially those with hypoxaemia. The proportion of hypoxaemic children identified by CLHWs with SpO_2_ <90% (1.2%) and those with SpO_2_ 90%–92% was a little lower than the proportion in our own study (figure 1 and table 3). Similarly, those with danger signs of pneumonia were also similar (<1%). Both hypoxaemic and danger sign pneumonia cases were excluded from enrolment in our study. However, the proportion of chest-indrawing patients in our community-based study was higher than that reported by the Malawi vaccine study for CLHWs.^35^ Another observational study from Malawi that linked community-based and health centre-based data with the hospital admission data reported that all deaths in the hospitalised pneumonia patients occurred within 24 hours of admission,^36^ which could be due to delay in appropriate care seeking.

### Interpretation and implications of the findings

There are a few potential reasons why the community-level treatment of children with chest-indrawing pneumonia was non-inferior to the facility-based treatment (standard iCCM protocols) and why we observed low treatment failure rates in both the intervention and control clusters. The treatment success depends on early recognition of pneumonia by the families or CLHWs, prompt care seeking from trained healthcare providers and timely and appropriate treatment. In our study, families sought early care from the trained CLHWs or community volunteers present in their communities, or they received home visits by them. The CLHWs in the intervention clusters provided prompt and appropriate treatment to the children with chest-indrawing pneumonia. The CLHWs in the control clusters immediately referred them to the referral level facilities after giving them a prereferral antibiotic dose. Thus, delayed referrals and failure to seek appropriate care, which are important determinants of treatment failure and pneumonia mortality, were avoided in both groups.^18 38^

Where access to health facilities is easy, and good quality care is available, management of chest-indrawing pneumonia by the CLHWs may not be necessary. However, in places where access is poor, including remote areas, difficult terrains, and humanitarian settings, treatment of chest-indrawing pneumonia and the use of pulse oximetry to assess hypoxaemia by CLHWs could potentially save lives. Treating chest-indrawing pneumonia at the community level has some potential advantages, including cost saving, ease for the families, getting treatment from someone the family knows and who is accessible and provides higher satisfaction than facility-based care.^39 40^ One cannot overlook the economic cost of out of pocket expenses for seeking care at the health facilities for treatment of pneumonia, which can be manifold higher than the community-level treatment cost.^41–43^

There are useful lessons from this trial for iCCM of pneumonia, diarrhoea and malaria programmes. First, sustaining the clinical skills of CLHWs over time is challenging,^44^ especially in settings where only a few sick children are seen weekly at the community level. Ongoing supervision, training and mentoring are required to ensure consistent quality of care.^45–48^ Second, a regular supply of essential commodities to avoid stockouts is imperative.^48^ Third, CLHWs accurately performed pulse oximetry to identify hypoxaemic children with pneumonia, who were referred immediately to a hospital for further management, and all survived up to 2 weeks of follow-up. When adequately trained, regularly monitored and supervised, and supplied with appropriate equipment (pulse oximeter, paediatric probes), CLHWs performed pulse oximetry correctly in children with chest-indrawing pneumonia. We selected the cut-off value for hypoxaemia (SpO_2_ <90%) as recommended by the WHO,^16^ although some data have been published about SpO_2_ between 90% and 93% as a risk factor for pneumonia mortality.^15 49^ Among the 10 deaths in this study, 9/10 (90%) had a SpO_2_ of 93%–100%, and 1 (10%) had an SpO_2_ between 90% and <93% (online supplemental Table 3). However, the low proportion (<2%) of hypoxaemia in children with chest-indrawing pneumonia seen in our study and elsewhere^34^ raises questions about the use of pulse oximetry at the community level. Implementation of pulse oximetry at the CLHW level is not inconsequential. It brings forth real issues regarding ensuring the quality with the use of pulse oximeter, selection of an appropriate pulse oximeter and age-specific probes and that it does not replace the clinical examination. Additionally, the health system support and scale-up requirements will require another level of engagement and investment by the policymakers and implementers. Further research is required in various geographic regions to evaluate the use of pulse oximeters by CLHWs, especially in resource-limited communities where access to a primary-level healthcare facility is poor. Finally, the existing large-scale iCCM programmes would need some implementation experience to learn about barriers and facilitators before scaling up the inclusion of chest-indrawing pneumonia treatment.

There are two major interlinked concerns with the empiric use of antibiotics to treat pneumonia at the community level. First, pneumonia in many of these children with lower chest-indrawing is non-bacterial that does not need an antibiotic, and second, the use of antibiotics by CLHWs may contribute to antimicrobial resistance.^50 51^ Until a good point of care, test is available to differentiate viral from bacterial infections, empiric antibiotic therapy is a useful tool to reduce pneumonia mortality. WHO standard case management of pneumonia promotes the rational use of antibiotics,^52 53^ which has also been demonstrated through CLHWs treatment of pneumonia in Zambia.^54^ To reduce antibiotic use and minimise antimicrobial resistance, more needs to be done. Recently, treatment with a shorter 3-day oral amoxicillin was shown to be non-inferior to a 5-day standard course in Malawian children with chest-indrawing at a health facility level.^55^ Further data are needed from other regions to validate these findings.

### Strengths and limitations

The strength of this trial is that this is a large multicountry trial that leveraged existing iCCM infrastructure across four African and Asian countries and demonstrated that the community-level treatment of children with chest-indrawing pneumonia is safe and feasible across a wide range of settings and seasons. Thus, these findings contribute to the body of evidence that CLHWs can effectively treat chest-indrawing pneumonia in low-resource settings, especially in areas where access to health centres is limited.^12–14^ Our trial also demonstrated the ability of CLHWs to use pulse oximetry successfully at the community level to make iCCM safer. Finally, the large sample size was adequate to detect a smaller non-inferiority margin, which was appropriate given the observed lower failure rate.

Our study had the limitation of using the WHO criterion of chest-indrawing pneumonia for diagnosis as the standard tools of radiology and microbiology were not feasible in the community setting. No single clinical sign can diagnose radiological pneumonia definitively,^56 57^ but in a meta-analysis, chest-indrawing along with respiratory rate higher than 50 bpm, grunting and nasal flaring, had the highest pooled estimates of the positive likelihood ratio for radiological pneumonia.^56^ On the other hand, determining the aetiology in physician-diagnosed hospitalised children with pneumonia is difficult even when multiple samples from multiples sites are tested in high-quality laboratories shown by the PERCH study.^58^ Thus, it is likely that our study included children with non-bacterial chest-indrawing pneumonia who were treated with antibiotics. However, because our primary outcome of treatment failure (excluding death) was only measured on day 6 of enrolment by independent outcome assessors, we believe that practically all children with only viral pneumonia would have gotten better by then and had minimal effect on the primary outcome. We enrolled a relatively higher number of children from Asian sites compared with African sites, instead of equal numbers. However, the subanalysis showed no difference in treatment failure rates between the intervention and control clusters in Asian and African sites.

## Conclusions

This large multicountry trial showed that trained, supervised and equipped with oral amoxicillin CLHWs can safely and effectively treat non-hypoxaemic 2–59-months-old children with chest-indrawing with a 5-day oral amoxicillin course in the community. CLHWs performed pulse oximetry correctly in most cases. Treatment of chest-indrawing pneumonia in children 2–59-months-old by CLHWs would increase access, particularly in settings where access to a health facility is limited and referral to a health facility is not feasible. This evidence should be used to review the current WHO/UNICEF iCCM protocol.

## Data Availability

Data are available upon reasonable request. Data are available upon request soon after publication.
