# Supplementary material for: Innovative, enhanced community management of non-hypoxaemic chest-indrawing pneumonia in 2–59-month-old children: a cluster-randomised trial in Africa and Asia
Source: BMJ Glob Health. 2022 Jan 5;7(1):e006405. doi: 10.1136/bmjgh-2021-006405 (PMC8734014; doi:10.1136/bmjgh-2021-006405)
Supplement: Supplementary data [file bmjgh-2021-006405supp001.pdf]

BMJ Publishing Group Limited (BMJ) disclaims all liability and responsibility arising from any reliance placed on this supplemental material which has been supplied by the author(s)

Supplementary material

Supplementary Table 1. Site characteristics by the study site

Characteristic	Bangladesh	Ethiopia	India	Malawi
Number of days a CLHW works in a week	6 days	5 days	6 days	3 days*
The average number of hours per day a CLHW works	6 hours between 9:00 AM to 3:00 PM	8 hours between 8:30 AM to 5:30 PM	6-7 hours between 9:00 AM to 5:00 PM	5-6 hours between 8.00 AM to 4:00 PM
CLHW works on the weekend or public holidays	No	No	Yes	Yes†
The proportion of families who take sick children directly to the primary health facility/Hospitals instead of going to the CLHW?	30-40%	65-70%	70%	No
‡Average time difference (in minutes) between CLHW clinical assessment SpO_2 measurement and re-assessment by the study supervisors	30 – 40 minutes	30 – 60 minutes	30 – 40 minutes	30 – 45 minutes

*3 days is minimum work in the community during daytime hours. The other two days, they work in the nearby health facility, so they actually work for 5 days. However, they are available 24 hours, including weekends.
†Not all CLHWs work on the weekend/public holiday
‡Occasionally it took up to two hours for a supervisor to reach the CLHW in areas with difficult terrain or during the rainy season.

. *BMJ Global Health* 2022; 7:e006405. doi: 10.1136/bmjgh-2021-006405

Supplementary Table 2. Definition of clinical signs

**Clinical sign**	**Definition**
Fast breathing	In children 2-11 months of age respiratory rate of 50 or more breaths per minute<br>In children 12-59 months old respiratory rate of 40 or more breaths per minute.
Chest indrawing	The lower chest wall (lower ribs) deeply and persistently moves in (retract) when the child takes a breath in (on inspiration).
Danger signs (Unable to feed or poor feeding on observation, vomit everything, convulsion or lethargic)	Ask mother:<br>Is the child able to drink or breastfeed?<br>Does the child vomit everything?<br>Has the child had convulsions?<br>Look:<br>If the child is lethargic or unconscious.<br>Is the child convulsing now?
Malnutrition	No malnutrition: MUAC >12.5 cm<br>Moderate malnutrition: MUAC from 11.5 to 12.5 cm<br>Severe malnutrition: MUAC <11.5 cm

. *BMJ Global Health* 2022; 7:e006405. doi: 10.1136/bmjgh-2021-006405

Supplementary Table 3. Danger signs identified by the CLHWs during screening (n=369)

Danger signs	Number (%)*
Cough for 14 days or more	75 (20.3%)
Diarrhoea for 14 days or more	16 (4.3%)
Blood in stools	16 (4.3%)
Fever (temperature 38°C or above) for seven days or more	42 (11.4%)
Convulsions or fits	12 (3.2%)
Difficult drinking or feeding	28 (7.6%)
Persistent vomiting	43 (11.6%)
Unusually sleepy or unconscious	16 (4.3%)
Severely malnourished as identified through mid-upper arm circumference (MUAC) <11.5 cm	163 (44.2%)
Swelling of both feet	17 (4.6%)

*The total is more than 369 because of multiple responses

. *BMJ Global Health* 2022; 7:e006405. doi: 10.1136/bmjgh-2021-006405

Supplementary Table 4. Follow-up of enrolled children in intervention clusters by CLHWs (n=2146)

Study site	Number of enrolled children	Follow-up after enrollment		
		Day 2, n (%)	Day 4, n (%)	Day 7, n (%)
Bangladesh	556	547 (98.4%)	539 (96.9%)	532 (95.7%)
Ethiopia	678	672 (99.1%)	665 (98.1%)	662 (97.6%)
India	684	678 (99.1%)	670 (97.9%)	667 (67.5%)
Malawi	228	216 (94.7%)	216 (94.7%)	10 (4.4%)
**Total**	**2146**	**2113 (98.5%)**	**2090 (97.4%)**	**1871 (87.2%)**

Supplementary Table 5. Characteristics of deaths among enrolled 2-59 months old children with chest indrawing pneumonia (n=10)

Characteristics	Intervention clusters (n=5)	Control clusters (n=5)
**At the time of enrolment** – no./total no. (%)		
Age (in months)		
2-11 months	4/5 (80%)	5/5 (100%)
12-59 months	1/5 (20%)	0/5 (0%)
Sex		
Male	1/5 (20%)	3/5 (60%)
Female	4/5 (80%)	2/5 (40%)
Mid upper arm circumference (MUAC)*		
MUAC Between 11.5 and 12.5 cm	3/5 (60%)	1/1 (100%)
MUAC >12.5 cm	2/5 (40%)	0/1 (0%)
Respiratory rate (breaths per minute)		
<60 breaths/minute	2/5 (40%)	3/5 (60%)
≥60 breaths/minute	3/5 (60%)	2/5 (40%)
Axillary temperature (°C)		
<38°C	3/5 (60%)	4/5 (80%)
≥38 °C	2/5 (40%)	1/5 (20%)
Oxygen saturation (SpO_2)		
90-<93%	1/5 (20%)	0/5 (0%)
93%-100%	4/5 (80%)	5/5 (100%)
Place of initiation of treatment at the time of enrolment		
Outpatient treatment	5/5 (100%)	4/5 (80%)
Inpatient treatment	0/5 (0%)	1/5 (20%)
**At the time of death** – no./total no. (%)		
Death occurred		
Up to 48 hours of enrolment	3/5 (60%)	1/5 (20%)
49 to 96 hours of enrolment	0/5 (0%)	2/5 (40%)
More than 96 hours of enrolment	2/5 (40%)	2/5 (40%)
Had any general danger sign† or hypoxaemia‡ at the	4/5 (80%)	5/5 (80%)
time of death	2/5 (40%)	2/5 (40%)
Place of death		
At home	2/5 (40%)	4/5 (80%)
At clinic/hospital	1/5 (20%)	1/5 (20%)
On the way to clinic/hospital	2/5 (40%)	0/5 (0%)

* Among children >=6 months of age

† General danger signs list: unable to feed or poor feeding on observation, convulsion, unusually sleepy or unconscious, vomit everything. In the intervention clusters, two children were unable to feed or had poor feeding, one child had convulsions, while one child was unable to feed or had poor feeding and hypoxaemia (SpO_2 <83% on day 4 and was referred to a hospital). One child had lower chest indrawing on day 6. In the control clusters, four children were unable to feed or had poor feeding, and one child was vomiting everything and unable to feed or had poor feeding.

‡ Hypoxaemia: SpO_2<90%

. *BMJ Global Health* 2022; 7:e006405. doi: 10.1136/bmjgh-2021-006405

Supplementary Table 6: Children with various oxygen saturation (SpO_2) categories by study sites (n=2255)

**Study site**	**Oxygen saturation category**			**Total**
	**<90%, n (%)**	**90-<93%, n (%)**	**93-100%, n (%)**	
Bangladesh	6 (1.0%)	25 (4.3%)	551 (94.7%)	582
Ethiopia*	27 (3.8%)	301 (42.3%)	384 (53.9%)	712
India	2 (0.3%)	12 (1.6%)	718 (98.1%)	732
Malawi	1 (0.4%)	17 (7.5%)	211 (92.1%)	229

*The Ethiopian study site is between 2100 and 2300 meters above the sea level compared to the other sites, which are mostly at or little above the sea level. This is probably the reason oxygen saturation levels in the Ethiopia site are in general lower than other sites.

. *BMJ Global Health* 2022; 7:e006405. doi: 10.1136/bmjgh-2021-006405
